# Advanced glycation endproducts are increased in rheumatoid arthritis patients with controlled disease

**DOI:** 10.1186/ar3538

**Published:** 2011-12-14

**Authors:** Lodewijk de Groot, Helmy Hinkema, Johanna Westra, Andries J Smit, Cees GM Kallenberg, Marc Bijl, Marcel D Posthumus

**Affiliations:** 1 Department of Rheumatology and Clinical Immunology, University Medical Centre, University of Groningen, Groningen, The Netherlands; 2 Department of Vascular Disease, University Medical Centre, University of Groningen, Groningen, The Netherlands

**Keywords:** rheumatoid arthritis, endothelial cell activation, endothelial dysfunction, *intima media *thickness, advanced glycation end products, atherosclerosis

## Abstract

**Introduction:**

Advanced glycation end products (AGEs) are produced and can accumulate during chronic inflammation, as might be present in patients with rheumatoid arthritis (RA). AGEs are involved in the development of cardiovascular disease. The aim of this study is to evaluate whether AGEs are increased in patients with long-standing RA and whether AGE accumulation is related to disease activity, disease severity and measures of (premature) atherosclerosis, such as endothelial activation, endothelial dysfunction and *intima media *thickness (IMT).

**Methods:**

In a cross-sectional study, 49 consecutive RA patients with longstanding disease (median disease duration of 12.3 years (range 9.3 to 15.1)), receiving standard of care, were included and compared with 49 age- and sex-matched healthy controls (HC). AGEs were determined by skin autofluorescence. Disease activity was evaluated by the Disease Activity Score of 28 joints (DAS-28) score and joint damage by modified Sharp-v.d. Heijde score. Endothelial activation (soluble vascular cellular adhesion molecule-1) sVCAM-1, von Willebrand factor (vWF), thrombomodulin), endothelial dysfunction (determined by small artery elasticity (SAE)) and IMT were measured and related to AGE accumulation.

**Results:**

AGEs were increased in RA patients (median 2.4 arbitrary units (a.u.), range 1.6 to 4.2) compared to HC (2.2, 1.3 to 3.8). RA patients had a DAS-28 score of 2.9 (0.8 to 6.9) and a modified Sharp-v.d. Heijde score of 19 (0 to 103). sVCAM-1 and vWF levels were higher in RA patients. SAE was significantly decreased in RA (3.9 ml/mmHg (1.4 to 12.2) vs. 6.1 in HC (1.7 to 12.9). IMT did not differ between the two groups. Combining both groups' AGEs correlated with vWF, sVCAM-1 and IMT, and was inversely related to SAE. In RA, AGEs had an inverse relation with SAE, but did not relate to disease activity or radiological damage. In multivariate analysis for both groups, smoking, glucose levels, vWF, SAE and male gender were significantly related to the formation of AGEs.

**Conclusions:**

AGEs were increased in RA patients with long-standing disease and without signs of premature atherosclerosis. AGEs were related to endothelial activation and endothelial dysfunction. This supports the hypothesis that in RA AGEs may be an early marker of cardiovascular disease.

## Introduction

Rheumatoid arthritis (RA) is associated with an excess morbidity and mortality due to cardiovascular disease (CVD). In a recent study, the risk for development of cardiovascular disease in RA was comparable with that in diabetics [1]. The excess in morbidity and mortality in RA patients due to CVD cannot be explained by traditional risk factors alone [2]. One of the non-traditional risk factors involved in patients with RA is considered to be chronic inflammation [3].

Chronic inflammation is supposed to accelerate the formation of the atherosclerotic plaque [4-6]. Inflammation results in endothelial activation and dysfunction, which are considered to be the first steps in the process finally resulting in atherosclerosis [7,8]. Endothelial cell activation is characterized by up-regulation and release of adhesion molecules, such as von Willebrand Factor (vWF), soluble vascular cell adhesion molecule-1 (sVCAM-1) and thrombomodulin (TM). Endothelial cell activation is followed by endothelial cell dysfunction. This phase is characterized by influx of inflammatory cells into the *intima *of the vascular wall and movement of smooth muscle cells out of the *tunica media *into the *intima*. Mononuclear cells absorb lipoproteins, such as oxidized LDL to form foam cells [6]. This process leads to an increased "stiffness" of the arterial wall, which can be measured by several methods, such as pulse wave analysis (PWA). PWA is a non-invasive method in which the elasticity of the radial artery is calculated by tonometry of the radial artery. PWA has been proven a valid method to identify endothelial dysfunction in RA [8]. Measurement of *intima media *thickness (IMT) serves as a surrogate marker for atherosclerosis [9].

Chronic inflammation might result in the production of advanced glycation end products (AGEs). Increased levels of AGEs are correlated with the development of future microvascular and macrovascular events in diabetics and non-diabetics [10-12]. AGEs can be quantified by a validated method that determines autofluorescence in the skin [13] and are found in atherosclerotic plaques [14]. AGEs can also be measured in plasma and urine. We deliberately chose measuring AGEs in the skin because AGEs in the skin reflect oxidative stress over a longer period of time compared to AGEs in plasma and urine, which reflect a more acute phase of oxidative stress. In a study in SLE patients, AGEs in the skin proved to be elevated in contrast to AGEs in plasma, which were not elevated [15]. AGEs are formed by cross-linking of proteins, nucleic acids and lipids [16] and can be a ligand for the receptor of AGE (RAGE), which is expressed on neutrophils, macrophages, T-cells and synovial fibroblasts [17]. Other known ligands for RAGE are, for example, HMGB1 and S100A12. Higher levels of HMGB1 are found in RA and predict mortality after myocardial infarction [18,19]. Increased levels of S100A12 are correlated with higher mortality in dialysis patients [20]. Ligation of AGE to RAGE results in NF-κB migration to the nucleus, stimulates transcription of pro-inflammatory genes and leads to up-regulation of endothelial adhesion molecules, such as sVCAM-1. sVCAM-1 expression facilitates adhesion of circulating T-lymphocytes [21]. As such, AGE-RAGE interaction can become a self-maintaining process, contributing to the development of atherosclerosis [22,23]. In RA, AGEs can be generated as a result of oxidative stress during inflammation.

We hypothesize that in longstanding RA, AGEs are increased due to prolonged exposure to oxidative stress and that AGE accumulation is related to endothelial activation, small artery elasticity and IMT, and to markers of disease damage. As such, AGEs might serve as a measure of cumulative inflammation and might be a predictor for CVD in RA patients.

## Materials and methods

### Patients and controls

Consecutive patients fulfilling the American College of Rheumatology (ACR) criteria for RA and having a disease duration of at least 9 years with a maximum of 15 years, who attended the outpatient clinic of the University Medical Center Groningen, were asked to participate in this study.

Exclusion criteria were pregnancy, diabetes mellitus (fasting blood glucose ≥7.0 mmol/L or the use of antidiabetic medications), renal impairment (serum creatinine > 140 μmol/L), surgery, myocardial infarction or sepsis in the past three months. Fifty patients were included. In addition, age- and sex-matched healthy volunteers were recruited as controls. After inclusion one patient declined further participation for personal reasons. The matched control was, therefore, also removed from further analysis.

Information was obtained from all subjects regarding traditional cardiovascular risk factors, including body mass index (BMI), smoking, blood pressure and lipid levels. Hypertension was defined as systolic blood pressure above 140 mmHg and/or a diastolic blood pressure above 90 mmHg and/or current use of antihypertensive drugs. Dyslipidaemia was defined as plasma cholesterol above 6.21 mmol/L, plasma LDL cholesterol above 3.36 mmol/L, plasma triglycerides above 2.26 mmol/L or current use of lipid lowering drugs. The study was approved by the local medical ethics committee of the University Medical Center of Groningen and informed consent was obtained from all study participants.

### Blood sampling and analysis

After an overnight fast, blood was sampled and creatinine, total cholesterol, triglycerides, high density lipoprotein (HDL), low density lipoprotein (LDL), C-reactive protein (CRP), erythrocyte sedimentation rate (ESR) and glucose were measured routinely. Additionally, serum and plasma samples were stored at -20°C for determination of endothelial cell activation markers. Serum levels of sVCAM-1 and thrombomodulin were measured by ELISA according to the manufacturer's instructions (R&D Systems, Abingdon, UK). Levels of vWF were determined using in-house ELISA as described [24].

### Measurement of disease activity

Disease activity was assessed using the Disease Activity Score for 28 joints (DAS-28 score) [25,26]. Ranges of DAS-28 correspond with disease activity. DAS-28 score < 2.6 indicates remission. DAS-28 score 2.6 to 3.2 indicates low disease activity. DAS-28 score 3.2 to 5.1 indicates moderate disease activity. DAS-28 score above 5.1 is considered high disease activity. Besides the DAS-28, for each patient the ACR/EULAR (American College of Rheumatology/European League Against Rheumatism) 2010 criteria for remission were determined. In these criteria patients are considered to be in remission if they have a maximum of 1 for each of the following: 28-joint count for swollen joints (SJC28) and tender joints (TJC28), CRP (mg/dl) and patient's global assessment (0 to 10 scale) [27].

### Measurement of functional disability

Traditionally, disability was assessed using the Health Assessment Questionnaire (HAQ), a score by questionnaire that examines the disabilities that RA-patients encounter in daily living and activities [11]. The final HAQ index ranges from 0 to 3. Although the mean HAQ of the population rises with age, HAQ scores < 0.3 are considered normal [28].

### Measurement of joint damage and cumulative CRP

To determine the amount of joint damage, we used the van der Heijde modification of the Sharp score. The maximum erosion score for the hands is 160 and for the feet 120; the maximum score for joint space narrowing is 120 for the hands and 48 for the feet, resulting in a maximum total score of 448 [29]. Radiography of the hands and feet were performed when recent radiographs, taken within one year, were not available. Cumulative CRP was calculated by calculating the area under the curve (AUC) by using the trapezoid model [30].

### Measurement of small artery elasticity

After overnight fasting, arterial elasticity was assessed using the CR-2000 (Hypertension Diagnostics, Eagan, MN, USA) for Pulse-Wave Analysis (PWA). The CR-2000 records and analyzes the blood pressure waveforms data from the Arterial Pulse Pressure Sensor. The distal elasticity of the small arteries (SAE) was estimated from a computerized pulse contour analysis [31,32]. The average of three readings of these parameters taken over 15 minutes was used. These measurements were performed on both the right and left radial artery. The average of the three measurements on each side were taken together and divided by two. Blood pressure was recorded as the average of three measurements on both arms by placing a blood pressure cuff on the opposite arm. Subjects lay in the supine position. For analysis, we used the mean systolic and diastolic blood pressure of these measurements.

### Measurement of intima-media thickness

Details of the method have been described by de Groot *et al*. [33]. In short, the IMT was determined in the far wall segments of the common carotid artery, carotid bulb and the internal carotid artery. A B-mode image of these segments was obtained from a lateral transducer position, during which the probe was positioned perpendicular to the far wall, showing an *intima-media *complex over approximately 1 cm. Subjects lay in the supine position. Mean IMT (the mean of the measurements in a segment) and maximum IMT (the highest IMT value found among the segment studied) per segment were calculated. Means of the mean IMT and means of the maximum IMT were calculated as the average for the six carotid segments of the mean value, and of the maximum value per segment, respectively. IMT was measured in a standardized way by the same experienced technicians. Coefficient of variation of IMT measurement of the GCA is approximately 5% [34].

### AGE measurement

AGEs were measured by using the auto-fluorescence reader (AGE-reader type 214B00102, DiagnOptics BV Groningen, The Netherlands, patent PCT/NL99/00607). The autofluorescence reader illuminates a skin surface of 4 cm^2^. Emission and excitation light from the skin are measured with a spectrometer. Patients and healthy controls were placed in a seated position and the reader was placed 10 cm below the elbow fold at the volar side. Autofluorescence is then calculated by dividing the average emitted light intensity per nanometer in the range of 420 to 600 nm by the average emitted light in the range of 300 to 420 nm [35]. When patients have visible sunburn no AGE measurement will be performed because of the fact that AGE values are falsely elevated in sunburned skin.

### Statistical methods

Data are expressed as median (range) unless stated otherwise. The sample size of 50 patients was calculated on detecting a difference of 0.4 arbitrary units (a.u.) in AGE level between RA and HC with a power of 80%, based on data from an earlier study by Lutgers *et al*. in diabetics [36]. Two-sample t-tests or Mann-Whitney-U tests were used as appropriate to make comparisons between patients and controls for continuous variables. For categorical variables the chi-square method and for very small expected frequencies the Fisher's exact test were used. Gaussian distribution of the data was analyzed with the D'Agostino-Pearson omnibus normality test. Correlation analysis was performed by Pearson correlation when variables were distributed normally; otherwise the Spearman correlation was used. Predictor analysis for AGE accumulation was performed using multivariate linear regression with forward inclusion of variables with *P *< 0.10 in univariate analysis. The probability of F for entry was 0.05, which means that variables were included until the *P*-value of the model did not improve anymore. Analyses were performed using Graphpad version 4.03 2005 (GraphPad Software, San Diego, California, USA) and SPSS version 14.0 (SPSS inc., Chicago, Illinois, USA). Two-sided *P*-values < 0.05 were considered significant.

## Results

### Clinical characteristics of patients and controls

Fifty RA patients and 50 healthy controls (HC) were included. One patient withdrew for personal reasons; subsequently, the matched control was excluded from analysis. Characteristics of patients and controls, including traditional cardiovascular risk factors, are shown in Table 1. RA patients had a less favorable cardiovascular risk profile: hypertension was more frequent in RA patients compared to HC. Both systolic and diastolic blood pressure were higher in patients. Furthermore, BMI and the percentage of active smokers were higher in the RA group. Although the proportion of individuals with dyslipidemia did not differ between RA and HC, absolute levels of HDL were lower and triglycerides were higher in RA patients (Table 1).

**Table 1:** Baseline characteristics of patients and controls

	RA patients N = 49	Controls N = 49	*P*-value
Age (years)	58 (31 to 74)	59 (29 to 75)	NS
Female (%)	61%	61%	NS
Hypertension (%)	42%	16%	0.012
Systolic blood pressure (mmHg)	140 (100 to 184)	130 (103 to 157)	0.016
Diastolic blood pressure (mmHg)	79 (55 to 113)	72 (60 to 96)	0.038
Body mass index (kg/m2)	26.6 (18.4 to 49.7)	24.5 (18.9 to 31.6)	0.002
Glucose (mmol/L)	5.3 (4.0 to 6.2)	5.2 (4.3 to 6.9)	NS
Creatinine (μmol/L)	73 (41 to 122)	71 (53 to 124)	NS
Dyslipidemia (%)	37%	35%	NS
Total cholesterol (mmol/L)	5.1 (2.9 to 7.1)	5.0 (2.4 to 6.8)	NS
High density lipoprotein (mmol/L)	1.4 (0.9 to 2.6)	1.7 (0.9 to 3.0)	0.004
Low density lipoprotein (mmol/L)	3.1 (1.6 to 5.3)	3.2 (1.6 to 4.9)	NS
Triglycerids (mmol/L)	1.14 (0.53 to 3.38)	0.95 (0.39 to 4.36)	0.047
Active smoking (%)	24%	10%	0.044

Hypertension was defined as: systolic blood pressure > 140 mmHg, diastolic blood pressure > 90 mmHg or use of antihypertensive drugs. Dyslipidemia was defined as: total cholesterol > 6.2 mmol/l, low density lipoprotein > 3.2 mmol/L, triglycerides > 2.26 mmol/L or use of lipid lowering drugs. All data are represented as median with range.

Disease characteristics of the RA patients are shown in Table 2. Disease activity as measured by DAS-28 score indicated generally minor disease activity, with normal distribution in D'Agostino and Pearson normality test. (median 2.9, range 0.8 to 6.9) and the modified Sharp-v.d. Heijde score was low (19 (0 to 103)) and was not normally distributed. Most RA patients were on disease-modifying antirheumatic drug (DMARD) therapy, the majority (63%) on methotrexate.

**Table 2:** Patient characteristics of 49 patients with RA

Disease duration (years)	12.3 (9.3 to 15.1) yrs.
Reumatoid factor positive (%)	100%
Anti-CCP positive	79%
DAS-28	2.92 (0.75 to 6.9)
Remission (ACR/EULAR 2010 criteria)	18%
HAQ score	0.18 (0.0 to 1.45)
Sharp van der Heijde score	19 (0 to 103)
Medication	MTX (63%)
	SSZ (16%)
	Anti-TNF (22%)
	Rituximab (2%)
	Leflunomide (6%)
	Azathioprine (3%)
	None (10%)

Disease duration in years. DAS-28 scores were normally distributed. HAQ and Sharp van der Heijde scores were not normally distributed.

### AGEs, endothelial cell activation, endothelial dysfunction and intima media thickness

**Figure 1:**
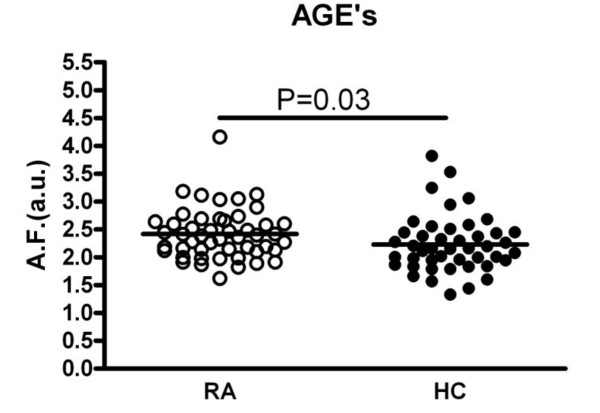
**AGE accumulation is significantly increased in RA patients in comparison to healthy controls**. The horizontal line denotes the median.

**Figure 2:**
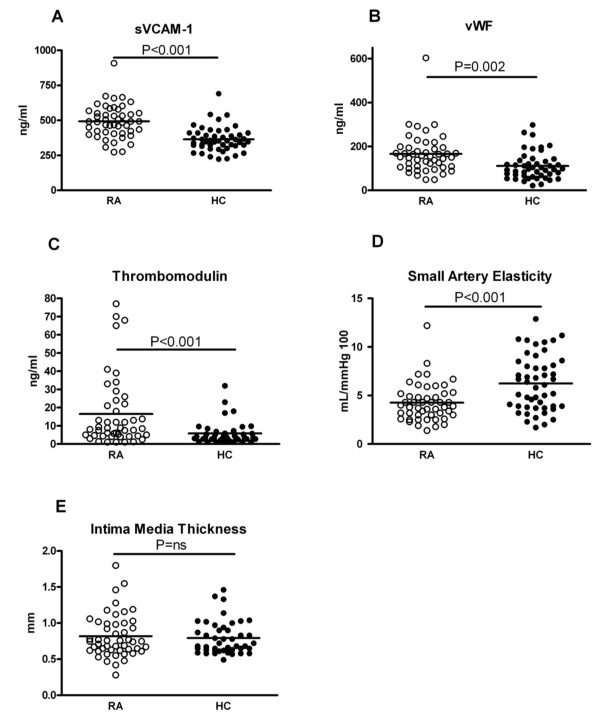
**Endothelial activation markers (sVCAM-1, vWF, thrombomodulin), endothelial dysfunction (SAE) and IMT in RA and HC**. sVCAM-1, panel A. vWF, panel B. thrombomodulin panel C. SAE, panel D. IMT panel E. Horizontal lines denote the median.

AGEs were increased (median 2.4 a.u., range 1.6 to 4.2) in this group of RA patients with longstanding disease in comparison to age- and sex-matched healthy controls (2.2 a.u., range 1.3 to 3.8) (Figure 1). Endothelial cell activation markers were increased in RA as well: sVCAM-1 (491 (range 274 to 909) vs. 354 ng/ml (range 224 to 691) in HC), vWF (154 (range 49 to 603) vs. 97 ng/ml (range 22 to 298) in HC) and thrombomodulin (8.5 (range 1 to 77) vs. 3.6 (range 1 to 32) ng/ml in HC) were all significantly elevated in patients (Figure 2). Endothelial dysfunction was present in RA as demonstrated by a decreased SAE of 3.9 (range 1.4 to 12.2) in patients vs. 6.1 (range 1.7 to 12.9) ml/mmHg in HC. In contrast, IMT did not differ between RA patients and HC (0.75 (range 0.28 to 1.80) vs. 0.70 mm (range 0.49 to 1.46) in HC) (Figure 2).

**Figure 3:**
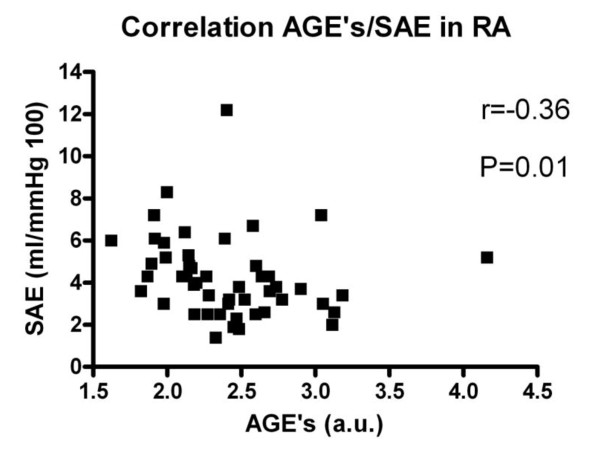
**Correlation between AGEs and SAE in RA**.

Additionally, in the total group (RA and HC) univariate analysis was performed (Table 3). AGE accumulation was not significantly related to the presence of RA (*P *= 0.06) but did show a significant association with smoking, creatinine, male gender, glucose, age, HDL cholesterol, triglycerides, systolic blood pressure, vWF and IMT. Furthermore, AGE accumulation was inversely related with SAE. In RA patients no correlation was found between AGEs and DAS-28 score, Sharp-vd Heijde score or cumulative CRP. In this group of 49 RA patients, also, no correlation was found between AGEs and markers of endothelial activation or IMT. However, in RA patients AGEs did show an inverse relation with SAE (Figure 3).

**Table 3:** Univariate and multiple linear regression analysis with AGEs as dependent variable.

	Univariate analysis		Multivariate analysis	
	B (95% C.I.)	*P*-value	B (95% C.I.)	*P*-value
R.A.	0.187 (-0.010 to 0.384)	0.063		
smoking	0.433 (0.194 to 0.672)	0.001	0.318 (0.120 to 0.516)	0.002
hypertension	0.135 (-0.069 to 0.339)	0.193		
BMI	0.011 (-0.010 to 0.032)	0.304		
dyslipidemia	0.124 (-0.084 to 0.331)	0.239		
creatinin	0.011 (0.006 to 0.017)	< 0.001		
Male gender	0.451 (0.266 to 0.637)	< 0.001	0.330 (0.262 to 0.508)	< 0.001
glucose	0.430 (0.247 to 0.612)	< 0.001	0.196 (0.022 to 0.370)	0.028
age	0.020 (0.011 to 0.028)	< 0.001		
total cholesterol	- 0.021 (-0.123 to 0.082)	0.688		
HDL-cholesterol	- 0.315 (-0.536 to 0.094)	0.006		
LDL-cholesterol	- 0.016 (-0.135 to 0.102)	0.784		
triglycerides	0.215 (0.056 to 0.374)	0.009		
RR systolic	0.007 (0.001 to 0.012)	0.030		
RR diastolic	0.010 (0.000 to 0.019)	0.046		
vWF	0.003 (0.001 to 0.004)	< 0.001	0.002 (0.001 to 0.003)	0.001
SAE	- 0.069 (-0.105 to -0.034)	< 0.001	- 0.035 (-0.069 to -0.002)	0.039
IMT	0.429 (0.136 to 0.722)	0.005		

Univariate and multiple linear regression analysis in both RA and HC cohorts combined. Multiple linear regression analysis was performed with forward inclusion of variables with P < 0.10 in univariate analysis. All data are represented as B with 95% confidence interval. The adjusted R-square of this model was 0.481.

### Multivariate analysis

In multivariate analysis with forward inclusion of variables with *P *< 0.10 as found in univariate analysis and F for entry 0.05, smoking, male gender, glucose level, vWF and SAE proved to be significant factors contributing to the formation of AGEs. (Table 3). The adjusted R square of this model was 0.481.

## Discussion

Premature atherosclerosis has been shown to be increased in RA [3,37,38]. Moreover, the presence of RA seems to be an independent risk factor for the development of CVD, equal to diabetes mellitus or smoking [1]. The key underlying mechanism is supposed to be the presence of chronic inflammation [6]. As chronic inflammation is represented by the accumulation of AGEs and AGEs contribute to the atherosclerotic process by themselves, in the current study we analyzed whether AGE accumulation occurs in RA patients with longstanding disease and whether AGE accumulation is related to markers of (premature) atherosclerosis and disease characteristics. In accordance with our findings in patients with other systemic autoimmune diseases [39,40], we found increased AGEs levels, as measured by skin auto-fluorescence, in RA patients.

AGEs are generated under the influence of oxidative stress as present in chronic inflammatory diseases like RA. By activating the receptor for AGE (RAGE), translocation of NF-κB to the nucleus is enhanced. In the nucleus, NF-κB facilitates the transcription of pro-inflammatory genes, finally resulting in, for example, the up-regulation of sVCAM-1. In this way a positive feed-back loop of AGE-RAGE interaction is established, maintaining an inflammatory status in which AGEs can be formed [17,21,41].

We found that AGEs were inversely related to SAE and positively related to IMT, suggesting that AGEs are involved in the formation of atherosclerotic plaques in RA. The process of atherosclerosis is assumed to start with endothelial activation [5]. Indeed, and in accordance with the results found by others, endothelial activation was increased in our patients, reflected by elevated sVCAM-1, vWF and thrombomodulin serum levels [42,43]. In addition, endothelial dysfunction, considered to be the next step in the atherosclerotic process, was present as shown by reduced SAE.

Although endothelial activation and endothelial cell dysfunction were present in our RA patients, IMT was not increased. Other studies, however, did find an increase in IMT in RA [44-50]. First, this discrepancy might be explained by differences in disease activity. In contrast to the patients included in our study, who had low disease activity (median DAS-28 of 2.92), the patients in the studies by Hannawi, Daza and Georgiadis had more active disease, represented by much higher DAS-28 scores of 4.39 (mean), 4.77 (median) and 5.8 (mean), respectively. During moderate to high disease activity edema of the vascular wall might be present. Therefore, it is possible that in patients with active RA and systemic inflammation, IMT not only reflects the presence of atherosclerotic plaques but also thickening of the *intima media *due to edema, leading to an overestimation of the IMT. Indeed, during follow-up Georgiadis *et al*. could demonstrate a reduction of IMT in RA patients, associated with a decrease in disease activity [45]. Also, in RA patients in remission (DAS-28 score below 2.60) with an average disease duration of three years, coronary artery disease was not increased compared to controls [51]. Secondly, discrepancies in IMT results might be explained by patient selection.

The RA patients included in this study, who were selected solely for having a long disease duration, are representative of our RA cohort and had increased prevalence of several traditional risk factors. This has been shown by others as well [48]. In particular, smoking is known as a risk factor not only for atherosclerosis, but also for RA [52]. In our study of the traditional risk factors for CVD, only smoking attributed to the accumulation of AGEs in multivariate analysis. In contrast to what might be expected, hypertension, BMI and dyslipidemia were not related to AGE accumulation.

Although AGEs were increased in our RA patients, factors other than the presence of RA seem to be more responsible for AGE accumulation. Multivariate analysis revealed that AGE accumulation was independently related to smoking, male gender, glucose level and serum vWF. In a study of 93 RA patients with a mean age of 61 years, AGEs proved to be increased compared to HC and osteoarthritis patients. In that study there was no correlation between AGE accumulation and severity of joint destruction, although there was a tendency to higher AGEs in more severe destructive disease [53]. We also did not find a relation between AGEs and disease severity, probably because joint damage in our cohort was quite low, indicating that RA patients in our cohort experienced low disease activity throughout the course of the disease. In a study with etanercept, in 22 RA patients with a mean disease duration of 15.3 years a significant reduction in serum and urinary levels of pentosidine (a sensitive marker for AGEs) was found after six months of treatment with etanercept. In this study, examining patients with high disease activity, a relation with decline in disease activity and pentosidine levels was found, indicating that disease activity is related to AGE formation [54]. In other auto-immune diseases, such as SLE, disease duration of more than 10 years was an independent predictor of AGE accumulation in the skin [39]. We speculate that in the RA patients included in the present study, low inflammatory activity throughout the course of the disease not only hampered occurrence of joint damage but also resulted in low production of AGEs and prevented an increase in IMT. Whether the presence of RA is an important factor in AGE accumulation in more active disease remains unclear and is the subject of future research.

## Conclusions

Our study shows increased AGE accumulation in RA patients, in particular related to smoking, male sex, glucose levels, endothelial activation and endothelial dysfunction. No relation was found between AGEs and actual disease activity and joint damage. Importantly, AGEs were inversely related to endothelial dysfunction in patients without signs of premature atherosclerosis. This supports the hypothesis that in RA AGEs may be early markers of cardiovascular disease. If so, this might open new therapeutic strategies with AGE breakers, such as alagebrium, a compound that not only degrades AGEs but also improves endothelial dysfunction in an animal model [55].

## Abbreviations

AF: Auto Fluorescence; AGEs: advanced glycation endproducts; Anti-CCP: anti-cyclic citrullinated protein; Anti-TNF: anti-tumour necrosis factor; a.u.: arbitrary units; BMI: body mass index; CRP: C-reactive protein; CVD: cardio vascular disease; DAS-28: disease activity score of 28 joins; DMARD: disease-modifying antirheumatic drug; ESR: erythrocyte sedimentation rate; EULAR: HAQ: health assessment quality; HC: Healthy Controls; HDL-cholesterol: high density lipoprotein cholesterol; IMT: *intima media *thickness; LDL-cholesterol: low density lipoprotein cholesterol; MTX: methotrexate; PWA: pulse wave analysis; RA: rheumatoid arthritis; RAGE: receptor for advanced glycation endproducts; RR diastolic: diastolic blood pressure; RR systolic: systolic blood pressure; SAE: small artery elasticity; sVCAM-1: soluble vascular cellular adhesion molecule-1; SSZ: sulfasalazine; TM: thrombomodulin; vWF: von Willebrand factor

## Competing interests

The authors declare that they have no competing interests.

## Authors' contributions

MP and HH participated in the design of the study. MP, HH, AS and JW participated in the acquisition of the data. LG, CK, MP and MB participated in the analysis and interpretation of the data and drafted the manuscript. All authors read and approved the final manuscript.

## References

[B1] van Halm VP Peters MJ Voskuyl AE Boers M Lems WF Visser M Stehouwer D Spijkerman AM Dekker JM Nijpels G Heine RJ Bouter LM Smulders YM Dijkmans BA Nurmohamed MT Rheumatoid arthritis versus diabetes as a risk factor for cardiovascular disease: a cross-sectional study, the CARRE investigation Ann Rheum Dis 2009 68 1395 1400 10.1136/ard.2008.094151 18697775

[B2] Van Doornum S McColl G Wicks IP Accelerated atherosclerosis: An extraarticular feature of rheumatoid arthritis? Arthritis Rheum 2002 46 862 873 10.1002/art.10089 11953961

[B3] del Rincon ID Williams K Stern MP Freeman GL Escalante A High incidence of cardiovascular events in a rheumatoid arthritis cohort not explained by traditional cardiac risk factors Arthritis Rheum 2001 44 2737 2745 10.1002/1529-0131(200112)44:12<2737::AID-ART460>3.0.CO;2-# 11762933

[B4] Quyyumi AA Inflamed joints and stiff arteries: Is rheumatoid arthritis a cardiovascular risk factor? Circulation 2006 114 1137 1139 10.1161/CIRCULATIONAHA.106.648139 16966595

[B5] Ross R Atherosclerosis--an inflammatory disease N Engl J Med 1999 340 115 126 10.1056/NEJM199901143400207 9887164

[B6] Libby P Ridker PM Maseri A Inflammation and atherosclerosis Circulation 2002 105 1135 1143 10.1161/hc0902.104353 11877368

[B7] Ridker PM Hennekens CH Roitman-Johnson B Stampfer MJ Allen J Plasma concentration of soluble intercellular adhesion molecule 1 and risks of future myocardial infarction in apparently healthy men Lancet 1998 351 88 92 10.1016/S0140-6736(97)09032-6 9439492

[B8] Van Doornum S McColl G Jenkins A Green DJ Wicks IP Screening for atherosclerosis in patients with rheumatoid arthritis: Comparison of two *in vivo *tests of vascular function Arthritis Rheum 2003 48 72 80 10.1002/art.10735 12528106

[B9] Duprez DA De Buyzere ML De Backer TL Veire VD Clement DL Cohn JN Relationship between arterial elasticity indices and carotid artery *intima-media *thickness Am J Hypertens 2000 13 1226 1232 10.1016/S0895-7061(00)01203-6 11078184

[B10] Gerrits EG Lutgers HL Kleefstra N Graaff R Groenier KH Smit AJ Gans RO Bilo HJ Skin autofluorescence: a tool to identify type 2 diabetic patients at risk for developing microvascular complications Diabetes Care 2008 31 517 521 18039805 10.2337/dc07-1755

[B11] Goh SY Cooper ME Clinical review: the role of advanced glycation end products in progression and complications of diabetes J Clin Endocrinol Metab 2008 93 1143 1152 10.1210/jc.2007-1817 18182449

[B12] Semba RD Ferrucci L Sun K Beck J Dalal M Varadhan R Walston J Guralnik JM Fried LP Advanced glycation end products and their circulating receptors predict cardiovascular disease mortality in older community-dwelling women Aging Clin Exp Res 2009 21 182 190 19448391 10.1007/bf03325227 PMC2684987

[B13] Mulder DJ Water TV Lutgers HL Graaff R Gans RO Zijlstra F Smit AJ Skin autofluorescence, a novel marker for glycemic and oxidative stress-derived advanced glycation endproducts: an overview of current clinical studies, evidence, and limitations Diabetes Technol Ther 2006 8 523 535 10.1089/dia.2006.8.523 17037967

[B14] Sakata N Imanaga Y Meng J Tachikawa Y Takebayashi S Nagai R Horiuchi S Increased advanced glycation end products in atherosclerotic lesions of patients with end-stage renal disease Atherosclerosis 1999 142 67 77 10.1016/S0021-9150(98)00192-0 9920507

[B15] Nienhuis HL de Leeuw K Bijzet J Smit A Schalkwijk CG Graaff R Kallenberg CG Bijl M Skin autofluorescence is increased in systemic lupus erythematosus but is not reflected by elevated plasma levels of advanced glycation endproducts Rheumatology (Oxford) 2008 47 1554 1558 10.1093/rheumatology/ken302 18701539

[B16] Smit AJ Lutgers HL The clinical relevance of advanced glycation endproducts (AGE) and recent developments in pharmaceutics to reduce AGE accumulation Curr Med Chem 2004 11 2767 2784 15544475 10.2174/0929867043364342

[B17] Pullerits R Bokarewa M Dahlberg L Tarkowski A Decreased levels of soluble receptor for advanced glycation end products in patients with rheumatoid arthritis indicating deficient inflammatory control Arthritis Res Ther 2005 7 R817 824 10.1186/ar1749 15987483 PMC1175032

[B18] Goldstein RS Bruchfeld A Yang L Qureshi AR Gallowitsch-Puerta M Patel NB Huston BJ Chavan S Rosas-Ballina M Gregersen PK Czura CJ Sloan RP Sama AE Tracey KJ Cholinergic anti-inflammatory pathway activity and high mobility group box-1 (HMGB1) serum levels in patients with rheumatoid arthritis Mol Med 2007 13 210 215 17597834 10.2119/2006-00108.Goldstein PMC1899837

[B19] Sorensen MV Pedersen S Mogelvang R Skov-Jensen J Flyvbjerg A Plasma high-mobility group box 1 levels predict mortality after ST-segment elevation myocardial infarction JACC Cardiovasc Interv 2011 4 281 286 10.1016/j.jcin.2010.10.015 21435605

[B20] Nakashima A Carrero JJ Qureshi AR Miyamoto T Anderstam B Barany P Heimburger O Stenvinkel P Lindholm B Effect of circulating soluble receptor for advanced glycation end products (sRAGE) and the proinflammatory RAGE ligand (EN-RAGE, S100A12) on mortality in hemodialysis patients Clin J Am Soc Nephrol 2010 5 2213 2219 10.2215/CJN.03360410 20847094 PMC2994082

[B21] Lander HM Tauras JM Ogiste JS Hori O Moss RA Schmidt AM Activation of the receptor for advanced glycation end products triggers a p21(ras)-dependent mitogen-activated protein kinase pathway regulated by oxidant stress J Biol Chem 1997 272 17810 17814 10.1074/jbc.272.28.17810 9211935

[B22] Kume S Takeya M Mori T Araki N Suzuki H Horiuchi S Kodama T Miyauchi Y Takahashi K Immunohistochemical and ultrastructural detection of advanced glycation end products in atherosclerotic lesions of human aorta with a novel specific monoclonal antibody Am J Pathol 1995 147 654 667 7545874 PMC1870970

[B23] Schleicher ED Wagner E Nerlich AG Increased accumulation of the glycoxidation product N(epsilon)-(carboxymethyl)lysine in human tissues in diabetes and aging J Clin Invest 1997 99 457 468 10.1172/JCI119180 9022079 PMC507819

[B24] de Leeuw K Sanders JS Stegeman C Smit A Kallenberg CG Bijl M Accelerated atherosclerosis in patients with Wegener's granulomatosis Ann Rheum Dis 2005 64 753 759 10.1136/ard.2004.029033 15374854 PMC1755479

[B25] van der Heijde DM 't Hof MA van Riel PL Theunisse LA Lubberts EW van Leeuwen MA van Rijswijk MH van de Putte LB Judging disease activity in clinical practice in rheumatoid arthritis: First step in the development of a disease activity score Ann Rheum Dis 1990 49 916 920 10.1136/ard.49.11.916 2256738 PMC1004262

[B26] Prevoo ML 't Hof MA Kuper HH van Leeuwen MA van de Putte LB van Riel PL Modified disease activity scores that include twenty-eight-joint counts. development and validation in a prospective longitudinal study of patients with rheumatoid arthritis Arthritis Rheum 1995 38 44 48 10.1002/art.1780380107 7818570

[B27] Jacobsson LT Hetland ML New remission criteria for RA: 'modern times' in rheumatology--not a silent film, rather a 3D movie Ann Rheum Dis 2011 70 401 403 10.1136/ard.2010.145607 21292832

[B28] Vita AJ Terry RB Hubert HB Fries JF Aging, health risks, and cumulative disability N Engl J Med 1998 338 1035 1041 10.1056/NEJM199804093381506 9535669

[B29] van der Heijde DM Plain X-rays in rheumatoid arthritis: Overview of scoring methods, their reliability and applicability Baillieres Clin Rheumatol 1996 10 435 453 10.1016/S0950-3579(96)80043-4 8876953

[B30] Crilly MA Kumar V Clark HJ Scott NW Macdonald AG Williams DJ Arterial stiffness and cumulative inflammatory burden in rheumatoid arthritis: a dose-response relationship independent of established cardiovascular risk factors Rheumatology (Oxford) 2009 48 1606 1612 10.1093/rheumatology/kep305 19858120

[B31] Finkelstein SM Cohn JN First- and third-order models for determining arterial compliance J Hypertens Suppl 1992 10 S11 14 10.1097/00004872-199204001-00003 1432309

[B32] Watt TB Jr Burrus CS Arterial pressure contour analysis for estimating human vascular properties J Appl Physiol 1976 40 171 176 1248996 10.1152/jappl.1976.40.2.171

[B33] de Groot E Zwinderman AH van der Steen AF Ackerstaff RG Montauban van Swijndregt AD Bom N Lie KI Bruschke AV Variance components analysis of carotid and femoral *intima-media *thickness measurements. REGRESS Study Group, Interuniversity Cardiology Institute of The Netherlands, Utrecht, The Netherlands. Regression Growth Evaluation Statin Study Ultrasound Med Biol 1998 24 825 832 10.1016/S0301-5629(98)00037-4 9740384

[B34] Sramek A Bosch JG Reiber JH Van Oostayen JA Rosendaal FR Ultrasound assessment of atherosclerotic vessel wall changes: reproducibility of *intima-media *thickness measurements in carotid and femoral arteries Invest Radiol 2000 35 699 706 10.1097/00004424-200012000-00001 11204795

[B35] Meerwaldt R Graaff R Oomen PH Links TP Jager JJ Alderson NL Thorpe SR Baynes JW Gans ROB Smit AJ Simple non-invasive assessment of advanced glycation endproduct accumulation Diabetologia 2004 47 1324 1330 15243705 10.1007/s00125-004-1451-2

[B36] Lutgers HL Graaff R Links TP Ubink-Veltmaat LJ Bilo HJ Gans RO Smit AJ Skin autofluorescence as a noninvasive marker of vascular damage in patients with type 2 diabetes Diabetes Care 2006 29 2654 2659 10.2337/dc05-2173 17130200

[B37] Avina-Zubieta JA Choi HK Sadatsafavi M Etminan M Esdaile JM Lacaille D Risk of cardiovascular mortality in patients with rheumatoid arthritis: A meta-analysis of observational studies Arthritis Rheum 2008 59 1690 1697 10.1002/art.24092 19035419

[B38] Wolfe F Mitchell DM Sibley JT Fries JF Bloch DA Williams CA Spitz PW Haga M Kleinheksel SM Cathy MA The mortality of rheumatoid arthritis Arthritis Rheum 1994 37 481 494 10.1002/art.1780370408 8147925

[B39] de Leeuw K Graaff R de Vries R Dullaart RP Smit AJ Kallenberg CG Bijl M Accumulation of advanced glycation endproducts in patients with systemic lupus erythematosus Rheumatology (Oxford) 2007 46 1551 1556 10.1093/rheumatology/kem215 17848401

[B40] de Leeuw K Nienhuis H Smit A Stegeman C Kallenberg C Bijl M Increased accumulation of advanced glycation endproducts in patients with Wegener's granulomatosis Ann Rheum Dis 2010 69 625 627 19854707 10.1136/ard.2009.123851

[B41] Carroll L Hannawi S Marwick T Thomas R Rheumatoid arthritis: links with cardiovascular disease and the receptor for advanced glycation end products Wien Med Wochenschr 2006 156 42 52 10.1007/s10354-005-0242-9 16465613

[B42] Foster W Carruthers D Lip GY Blann AD Inflammatory cytokines, endothelial markers and adhesion molecules in rheumatoid arthritis: effect of intensive anti-inflammatory treatment J Thromb Thrombolysis 2010 29 437 442 10.1007/s11239-009-0370-y 19578810

[B43] Pemberton PW Ahmad Y Bodill H Lokko D Hider SL Yates AP Walker MG Laing I Bruce IN Biomarkers of oxidant stress, insulin sensitivity and endothelial activation in rheumatoid arthritis: a cross-sectional study of their association with accelerated atherosclerosis BMC Res Notes 2009 2 83 10.1186/1756-0500-2-83 19426539 PMC2687454

[B44] Gonzalez-Juanatey C Llorca J Martin J Gonzalez-Gay MA Carotid *intima-media *thickness predicts the development of cardiovascular events in patients with rheumatoid arthritis Semin Arthritis Rheum 2009 38 366 371 10.1016/j.semarthrit.2008.01.012 18336869

[B45] Georgiadis AN Voulgari PV Argyropoulou MI Alamanos Y Elisaf M Tselepis AD Drosos AA Early treatment reduces the cardiovascular risk factors in newly diagnosed rheumatoid arthritis patients Semin Arthritis Rheum 2008 38 13 19 10.1016/j.semarthrit.2007.09.008 18191989

[B46] Kumeda Y Inaba M Goto H Nagata M Henmi Y Furumitsu Y Ishimura E Inui K Yutani Y Miki T Shoji T Nishizawa Y Increased thickness of the arterial *intima-media *detected by ultrasonography in patients with rheumatoid arthritis Arthritis Rheum 2002 46 1489 1497 10.1002/art.10269 12115178

[B47] Del Rincon I Freeman GL Haas RW O'Leary DH Escalante A Relative contribution of cardiovascular risk factors and rheumatoid arthritis clinical manifestations to atherosclerosis Arthritis Rheum 2005 52 3413 3423 10.1002/art.21397 16255018

[B48] Daza L Aguirre M Jimenez M Herrera R Bollain JJ Common carotid *intima-media *thickness and von Willebrand factor serum levels in rheumatoid arthritis female patients without cardiovascular risk factors Clin Rheumatol 2007 26 533 537 10.1007/s10067-006-0338-7 16758372

[B49] Park YB Ahn CW Choi HK Lee SH In BH Lee HC Nam CM Lee SK Atherosclerosis in rheumatoid arthritis: morphologic evidence obtained by carotid ultrasound Arthritis Rheum 2002 46 1714 1749 10.1002/art.10359 12124853

[B50] Hannawi S Haluska B Marwick TH Thomas R Atherosclerotic disease is increased in recent-onset rheumatoid arthritis: a critical role for inflammation Arthritis Res Ther 2007 9 R116 10.1186/ar2323 17986352 PMC2246234

[B51] Ma NH Teh CL Rapaee A Lau KB Fong AY Hi S Chang BC Yew KL Liew HB Ang CK Ong TK Chua SK Chin RW Sim KH Subclinical coronary artery disease in Asian rheumatoid arthritis patients who were in remission: a pilot study Int J Rheum Dis 2010 13 223 229 20704618 10.1111/j.1756-185X.2010.01533.x

[B52] Kallberg H Ding B Padyukov L Bengtsson C Ronnelid J Klareskog L Alfredsson L Smoking is a major preventable risk factor for rheumatoid arthritis: estimations of risks after various exposures to cigarette smoke Ann Rheum Dis 2011 70 508 511 10.1136/ard.2009.120899 21149499 PMC3033966

[B53] Matsumoto T Tsurumoto T Baba H Osaki M Enomoto H Yonekura A Shindo H Miyata T Measurement of advanced glycation endproducts in skin of patients with rheumatoid arthritis, osteoarthritis, and dialysis-related spondyloarthropathy using non-invasive methods Rheumatol Int 2007 28 157 160 10.1007/s00296-007-0408-4 17653550

[B54] Kageyama Y Takahashi M Nagafusa T Torikai E Nagano A Etanercept reduces the oxidative stress marker levels in patients with rheumatoid arthritis Rheumatol Int 2008 28 245 251 10.1007/s00296-007-0419-1 17661050

[B55] Soro-Paavonen A Zhang WZ Venardos K Coughlan MT Harris E Tong DC Brasacchio D Paavonen K Chin-Dusting J Cooper ME Kaye D Thomas MC Forbes JM Advanced glycation end-products induce vascular dysfunction via resistance to nitric oxide and suppression of endothelial nitric oxide synthase J Hypertens 2010 28 780 788 10.1097/HJH.0b013e328335043e 20186099

